# Quantification of hypoxia-related gene expression as a potential approach for clinical outcome prediction in breast cancer

**DOI:** 10.1371/journal.pone.0175960

**Published:** 2017-04-21

**Authors:** Abderrahim El Guerrab, Anne Cayre, Fabrice Kwiatkowski, Maud Privat, Jean-Marc Rossignol, Fabrice Rossignol, Frédérique Penault-Llorca, Yves-Jean Bignon

**Affiliations:** 1 Centre Jean Perrin, 58 rue Montalembert, Clermont-Ferrand, France; 2 Université d’Auvergne, ERTICa-EA4677, Faculté de Médecine, 28 place Henri Dunant, Clermont-Ferrand, France; 3 Adelbio, 13 Rue du Pariou, Aubière, France; University of North Carolina at Chapel Hill School of Medicine, UNITED STATES

## Abstract

Breast cancers are solid tumors frequently characterized by regions with low oxygen concentrations. Cellular adaptations to hypoxia are mainly determined by “hypoxia inducible factors” that mediate transcriptional modifications involved in drug resistance and tumor progression leading to metastasis and relapse occurrence. In this study, we investigated the prognostic value of hypoxia-related gene expression in breast cancer. A systematic review was conducted to select a set of 45 genes involved in hypoxia signaling pathways and breast tumor progression. Gene expression was quantified by RT-qPCR in a retrospective series of 32 patients with invasive ductal carcinoma. Data were analyzed in relation to classical clinicopathological criteria and relapse occurrence. Coordinated overexpression of selected genes was observed in high-grade and HER2+ tumors. Hierarchical cluster analysis of gene expression significantly segregated relapsed patients (p = 0.008, Chi^2^ test). All genes (except one) were up-regulated and six markers were significantly expressed in tumors from recurrent patients. The expression of this 6-gene set was used to develop a basic algorithm for identifying recurrent patients according to a risk score of relapse. Analysis of Kaplan-Meier relapse-free survival curves allowed the definition of a threshold score of 2 (p = 0.021, Mantel-Haenszel test). The risk of recurrence was increased by 40% in patients with a high score. In addition to classical prognostic factors, we showed that hypoxic markers have potential prognostic value for outcome and late recurrence prediction, leading to improved treatment decision-making for patients with early-stage invasive breast cancer. It will be necessary to validate the clinical relevance of this prognostic approach through independent studies including larger prospective patient cohorts.

## Introduction

Breast cancer is a heterogeneous disease with diverse clinical outcomes. Current therapeutic options, including initial surgery and both adjuvant chemotherapy and endocrine therapy, are effective in the earlier stages of disease and improve clinical outcome. However, despite the proven benefits of these treatments, breast cancer patients still have a risk of relapse after the first 5 years. The clinical outcome of breast cancer is based primarily on clinicopathological criteria such as tumor size, histological grade and the status of estrogen, progesterone and HER2 receptors. These parameters are prognostic markers for early recurrence, but their role in late recurrence is less clear [1, 2]. Prediction of late recurrence at diagnosis could help individualize therapeutic options, thereby preventing unnecessary treatments. Identification of factors predicting long-term relapse-free survival in breast cancer patients has become an important promising field of biomarker research [3].

Accumulating evidence from clinical studies suggests that tumor hypoxia might have an important role for clinical outcome and late recurrence in human cancer, including invasive breast cancer. The hypoxic tumor microenvironment is associated with a poorer prognosis for outcome and survival [4]. Several authors have shown that molecular mechanisms of adaptation to hypoxia make tumor cells more aggressive and more resistant to chemotherapy and radiotherapy, thereby promoting tumor progression [5, 6]. Hypoxic areas arise when the metabolic requirements of cancer cells are higher than the availability of intravascular oxygen content in tumors. Cellular adaptations to hypoxia are mainly mediated by a family of transcription factors called hypoxia inducible factors (HIFs). HIF-1 was the first member of this family and is ubiquitously expressed [7]. These transcription factors are heterodimers composed of an alpha subunit and a beta subunit [8]. Under normoxic conditions, HIF-1 alpha is hydroxylated by a family of dioxygenases known as prolyl-hydroxylases (PHD). Hydroxylated proline residues are recognized by the Von Hippel-Lindau tumor suppressor, leading to polyubiquitination and subsequent proteasomal degradation. Under hypoxic conditions, oxygen levels are not sufficient for the enzymatic activation of PHD. Consequently, HIF-1 alpha is not degraded and is translocated to the nucleus, where it binds to the subunit HIF-1 beta and the transcriptional coactivator p300 [9]. The active transcription complex regulates the expression of multiple genes by binding specific DNA sequences called hypoxia response elements (HRE). Regulation of HIF-1 alpha protein is not limited to hypoxic conditions. Several studies have also revealed oxygen-independent mechanisms that result from genetic alterations such as activation of oncogenes (*HER2*) and/or loss of tumor suppressor genes (*VHL* or *PTEN*). Dysfunctions of the PI3K/AKT and RAS/MAPK signaling pathways are also involved in HIF-1 alpha regulation [10, 11]. Activation of hypoxia-related genes plays an important role in tumor progression because of the involvement of these genes in several cellular processes, including cell differentiation, survival, angiogenesis, migration and metastasis [12].

Thus, assessment of tumor hypoxia appears to be a potential strategy for clinical outcome prediction of solid tumors. However, it remains difficult to perform quantitative measures of tumor hypoxia as well as to determine the relationship between hypoxia and clinical parameters in human cancers. Several measurement methods of tumor oxygenation, including both direct and indirect approaches, have been described. The main direct approach for measuring the partial pressure of oxygen in tumors is based on the polarographic method using oxygen microelectrodes. This method revealed a mean partial oxygen pressure of 28 mmHg in breast tumors and 65 mmHg in normal breast tissue [13]. Indirect methods consist essentially of immunohistochemical measurement in tumor biopsies of the expression of HIF-1 alpha as well as proteins regulated by HIF complexes, such as carbonic anhydrase 9 (CA9) and vascular endothelial growth factor (VEGF). Several previous reports have already associated breast cancer outcomes with levels of HIF-1 alpha or CA9 proteins [14–16]. Other non-invasive techniques, such as molecular imaging, allow the identification of intratumoral hypoxia by analyzing its effect on the metabolism of tumor cells. The low oxygen pressures observed in solid tumors force cells to shift from aerobic to anaerobic glucose metabolism [17]. Positron emission tomography (PET) imaging with 18F-fluorodeoxyglucose (18F-FDG) permits the detection of increased glucose consumption by cancer cells. 18F-FDG uptake correlates with reduced partial pressure of oxygen and increased HIF-1 alpha protein levels in diverse types of tumors [18, 19]. More recently, the analysis of changes induced by hypoxia in the transcriptome has also provided an indirect method with prognostic and predictive values [12]. Several molecular signatures have been constructed from non-specific genetic markers of hypoxic responses and breast cancer. Most of these signatures were generated by differential strategies based on whole-transcriptome analysis or were implemented initially from other solid tumors [20, 21]. Winter *et al*. defined a molecular signature of 99 genes whose expression in a series of head and neck squamous cell carcinomas clustered with the expression of 10 well-known hypoxia-regulated genes. This signature was shown to be a prognostic factor for relapse-free survival in an independent breast cancer series [20].

These studies highlight the importance of hypoxia-related gene expression for outcome prediction in breast cancer. The quantification of biomarkers involved in both hypoxia signaling pathways and breast cancer development may facilitate the prediction of prognosis according to the molecular profile of tumors. The aim of this study was to generate a molecular signature of tumor hypoxia with potential prognostic significance in breast cancer. We analyzed the expression of 45 well-known hypoxia-regulated genes in a retrospective series of 32 tumor samples from patients with early-stage invasive breast cancer. This set of genes was selected from a systematic review according to objective criteria based on their implication in breast cancer aggressiveness and hypoxia signaling pathways. Gene expression was investigated in relation to clinicopathological data (stage, grade mSBR, HER2 status, and relapse occurrence).

## Materials and methods

### Patients and clinicopathological data

A retrospective study of a total of 32 patients with previously untreated primary breast cancer was conducted. Patients were diagnosed between 1994 and 1998 and had undergone surgery at the Jean Perrin Comprehensive Cancer Center. Fine-needle aspiration biopsies were performed in patients, and an aliquot of each aspirate was immediately smeared on a slide to serve as a control for the presence of malignant cells and the absence of important stromal and fat contamination. The remaining aspirated material was processed for embedding in a paraffin block for later use in immunophenotyping or stored in liquid nitrogen until total RNA extraction. Tumors samples were conserved in the Biological Resource Center of Jean Perrin Comprehensive Cancer Center, identified under No. BB-0033-00075 (Clermont-Ferrand, France). The clinical history of patients was collected with the help of an oncologist. Tumors were classified histologically according to the World Health Organization criteria as ductal invasive breast carcinoma. Initial staging comprised complete and detailed clinical examination including the International Union Against Cancer TNM (tumor size, nodes, metastases) classification. Histopathological evaluation of tumors was performed using the Scarff-Bloom-Richardson histologic grading system as modified by Le Doussal [22, 23]. Under French law on biomedical research, this is an epidemiological study that does not have to be submitted to an Institutional Review Board. All clinical data and tissue samples were fully anonymized and de-identified before they were accessed by the researchers for this study.

### Immunohistochemical studies

Patients were screened for estrogen, progesterone and HER2 receptor status by immunohistochemistry (IHC) on paraffin-embedded tissue sections. Immunostaining was performed with a Nexes automated immunostainer following the manufacturer's guidelines (Ventana, Illkirch, France). Sections were scored semiquantitatively by two pathologists using standard light-microscopic evaluation. A threshold of 10% total stained tumor cells was considered positive for estrogen and progesterone status. Immunohistochemical staining for HER2 was performed using the HercepTest kit (Dako, Carpinteria, CA, USA) and was scored according to the standard scoring system recommended by the manufacturer. Intensity scores of 0 or 1+ were designated as negative for HER2 expression. Scores of 3+ were considered positive and were defined as HER2 overexpression in the presence of complete membrane staining with high intensity. Scores of 2+ were considered equivocal cases, and HER2 fluorescence *in situ* hybridization (FISH) assay was performed for detection of *HER2* amplification using the HER2 FISH pharmDx kit (Dako) according to the manufacturer's instructions. Tumors with amplification of *HER2* were considered HER2 positive (3+). Patient and tumor characteristics are summarized in Table 1.

**Table 1:** Clinical and histopathological characteristics of patients.

Characteristics	Classification	All patients (n = 32)
**Age**	< 50 ≥ 50	n = 8 n = 24
**Estrogen receptors**	Negative Positive	n = 1 n = 31
**Progesterone receptors**	Negative Positive	n = 8 n = 24
**Lymph nodes**	Negative Positive	n = 20 n = 12
**Tumor stage**	1 2–3	n = 7 n = 25
**Grade mSBR**	1-2-3 4–5	n = 25 n = 7
**HER2 status**	Negative Positive	n = 27 n = 5
**Recurrence**	No Yes	n = 18 n = 14

### RNA extraction and reverse transcription

Total RNA was extracted from frozen tumor samples using Trizol reagent according to the manufacturer’s protocol (Invitrogen Life Technologies, Carlsbad, CA, USA). The quality and concentration of the total RNA were assessed using an Agilent 2100 Bioanalyzer (Agilent Technologies, Foster City, CA, USA). Two micrograms of total RNA were reverse transcribed in a total volume of 20 μl using the High Capacity cDNA kit with RNase inhibitor according to the manufacturer’s instructions (Applied Biosystems, Foster City, CA, USA). The reaction conditions were 25°C for 10 min, 37°C for 120 min and 85°C for 5 min.

### Assay design and real-time quantitative PCR

A qualitative review of literature on breast cancer was performed in PubMed/MEDLINE to select 45 genes known to be regulated by hypoxia and involved in breast carcinogenesis. Selection of these genes was performed according to several criteria, including the presence of HRE elements in promoters, ability to be activated by hypoxia and/or hypoxia-mimetic agents such as desferrioxamine or cobalt chloride, and involvement in breast cancer aggressiveness (Table 2). Real-time quantitative PCR analysis was performed using custom-made Taqman low-density arrays (TLDAs), which are 384-well microfluidic cards preloaded with sets of primers and specific probes designed to amplify selected genes (Applied Biosystems, Foster City, CA, USA). Samples of cDNA (50 μl) were mixed with 50 μl of 2X Taqman Universal PCR Master Mix (Applied Biosystems), and a total of 100 μl of reaction mixture was loaded on TLDA cards, followed by centrifugation twice 1 min at 1200 rpm to distribute the samples from the loading port into each well. The cards were sealed, and real-time quantitative PCR amplification was performed using an ABI Prism 7900 HT Sequence Detection System according to the manufacturer's instructions (Applied Biosystems). Relative quantification (RQ) analysis was performed with RQ Manager 1.2 software (Applied Biosystems). A threshold cycle (Ct) value equal to 35 was used as the cutoff for non-expressed genes. The set of genes included two housekeeping genes used as internal controls (*RPL32* and *18S*). In addition, gene expression stability was determined by the NormFinder program, and optimal reference genes for normalization were identified among the selected genes [24]. The average expression level of all markers was also used to perform data normalization. The RQ of gene expression was determined using the comparative ΔΔCt method based on the equation RQ = 2^-ΔΔCt^ [25]. This method allows the determination of the relative fold change ratio of a target gene between two different groups.

**Table 2:** List of selected gene expression assays.

Gene symbol	Assay reference	Gene name
***Endogenous genes***		
*18S*	Hs99999901_s1	-
*RPL32*	Hs00851655_g1	Ribosomal protein L32
***Cell survival*, *proliferation*, *differentiation***		
*BNIP3*	Hs00969291_m1	BCL2/adenovirus E1B 19 kd-interacting protein 3
*BRCA1*	Hs00173233_m1	Breast cancer 1
*CCND1*	Hs00277039_m1	Cyclin D1
*EPO*	Hs01071096_g1	Erythropoietin
*HER2*	Hs01001595_m1	Erythroblastic leukemia viral oncogene homolog 2
*IGF2*	Hs01005964_g1	Insulin-like growth factor 2
*NDRG1*	Hs00608387_m1	N-myc downstream regulated gene 1
*BNIP3L*	Hs00188949_m1	BCL2/adenovirus E1B 19kDa interacting protein 3-like
*TGFB3*	Hs00234245_m1	Transforming growth factor beta
*TGM2*	Hs00190278_m1	Transglutaminase 2
***Transcription factors and feed back***		
*CEBPA*	Hs00269972_s1	CCAAT/Enhancer binding protein alpha
*CITED2*	Hs00366696_m1	Cbp/p300-interacting transactivator, 2
*ETS1*	Hs00901425_m1	v-ets erythroblastosis virus E26 oncogene homolog 1
*FOXO3A*	Hs00921424_m1	Forkhead box O3
*NR4A1*	Hs00374230_m1	Nuclear receptor subfamily 4, group A, member 1
*PHD2*	Hs00254392_m1	HIF-prolyl hydroxylase 2
*SNAI1*	Hs00195591_m1	Snail homolog 1
*TWIST1*	Hs00361186_m1	Twist homolog 1
*VHL*	Hs00184451_m1	Von Hippel-Lindau
*PTEN*	Hs00829813_s1	Phosphatidylinositol-3,4,5-trisphosphate 3-phosphatase
***Extracellular matrix*, *motility***		
*CTSD*	Hs00157201_m1	Cathepsin D
*CDH1*	Hs01023895_m1	E-cadherin
*KRT19*	Hs00761767_s1	Keratin 19
*CTGF*	Hs01026926_g1	Connective tissue growth factor
*CXCR4*	Hs00607978_s1	Chemokine (C-X-C motif) receptor 4
*MET*	Hs01565582_g1	The proto-oncogene MET
*MMP2*	Hs00234422_m1	Matrix metallopeptidase 2
*PLAUR*	Hs00182181_m1	Plasminogen activator, urokinase receptor
*VIM*	Hs00185584_m1	Vimentin
***Glucose metabolism*, *pH***		
*GPI*	Hs00976711_m1	Glucose phosphate isomerase
*CA9*	Hs00154208_m1	Carbonic anhydrase 9
*ENO1*	Hs00361415_m1	Enolase 1
*GLUT1*	Hs00892681_m1	Glucose transporter 1
*LDHA*	Hs00855332_g1	Lactate dehydrogenase A
*NHERF1*	Hs00188594_m1	Na/H exchanger regulatory factor 1
*PGK1*	Hs00943178_g1	Phosphoglycerate kinase 1
*TPI*	Hs01593134_gH	Triose-phosphate isomerase
***Angiogenesis***		
*COX2*	Hs01573471_m1	Cyclo-oxygenase 2
*EDN1*	Hs00174961_m1	Endothelin
*ENG*	Hs00164438_m1	Endoglin
*LEP*	Hs00174877_m1	Leptin
*VEGF*	Hs00900054_m1	Vascular endothelial growth factor
***Drug resistance***		
*AK3*	Hs00750261_s1	Adenylate Kinase 3
*ABCB1*	Hs01067802_m1	ATP-binding cassette, sub-family B member 1
*ABCG2*	Hs01053790_m1	ATP-binding cassette, sub-family G member 2

### Statistical analysis

Different groups of patients were defined according to clinicopathological criteria such as tumor stage, histological grade, HER2 status and occurrence of relapse. For each gene, the average RQ was calculated in each group. The ratio of the average RQ between 2 groups was used to determine the fold induction for the expression of each gene in a group of patients relative to the corresponding control group. A positive fold change of 1 indicated 2-fold up-regulation, and a negative fold change of -1 indicated 2-fold down-regulation. A comparative analysis of gene expression profiles was performed between different groups. A parametric (Student's t-test) or a non-parametric test (Kruskal-Wallis test) was used to identify genes that were significantly differently expressed between groups.

Unsupervised hierarchical clustering analysis based on ΔCt values was performed using the Euclidean distance and Ward’s method based on barycenter calculation. Gene expression profiles were analyzed using all selected genes and differentially expressed genes with statistical significance between the recurrent group and non-recurrent group. Secondary to cluster calculation, the Chi^2^ test was used to compare the proportion of relapses in the main selected clusters of patients. This approach permits the validation of the relevance of the cluster analysis and the influence of the expression of the genes on relapse risk.

Kaplan-Meier survival curves were constructed for distant or local relapse-free survival, and statistical significance was examined using the Mantel-Haenszel test. Relapse-free survival was defined as the time of diagnosis to the development of distant or local recurrence. The internal consistency of predictive markers of relapse was assessed using Cronbach’s alpha coefficient as a measure of scale reliability. All analyses were performed using the SEM statistical software [26], and a probability value p < 0.05 was considered significant.

## Results

### Comparative analysis of hypoxia-related gene expression according to clinicopathological data

**Fig 1:**
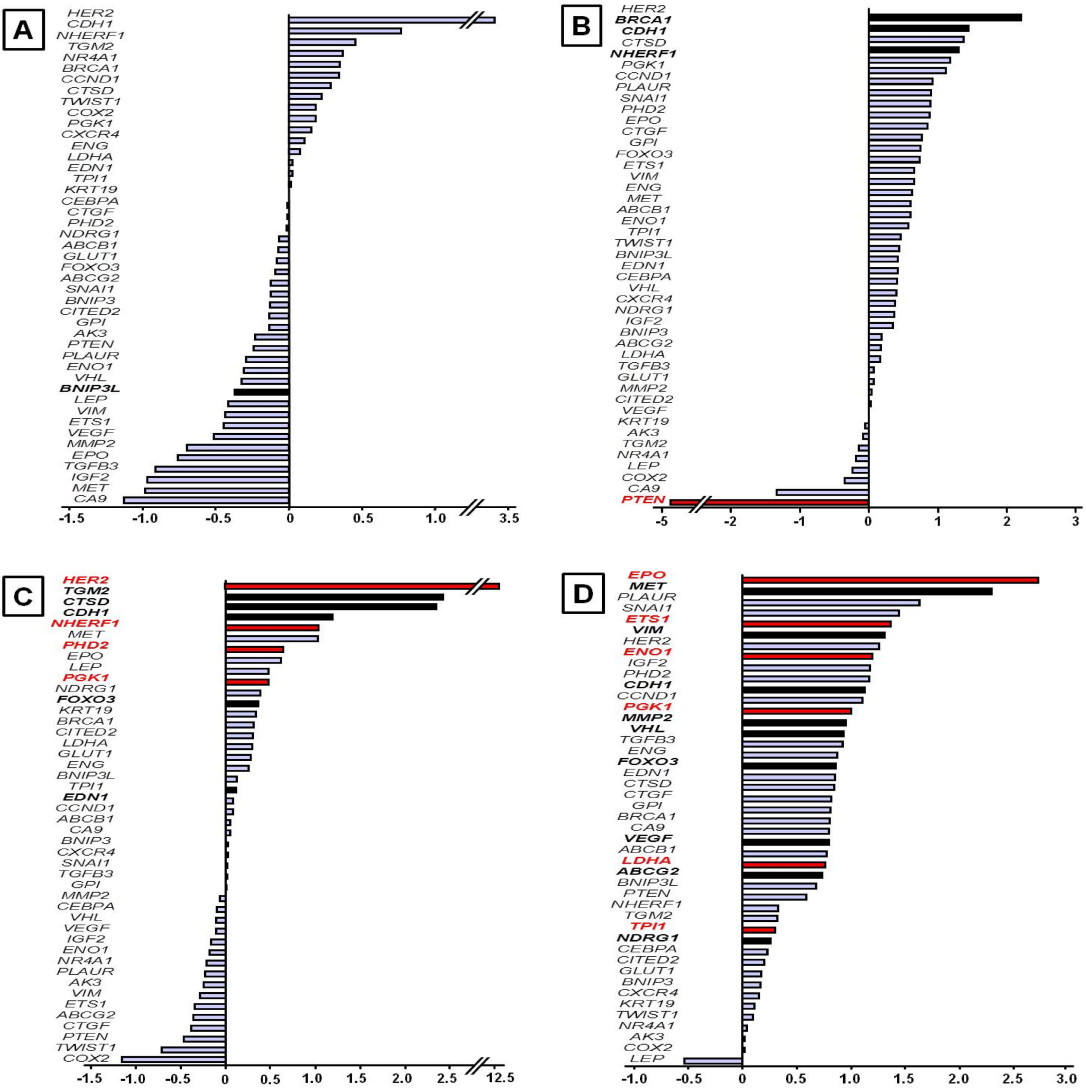
Hypoxia-related gene expression profiles according to clinicopathological data. Gene expression was determined using quantitative real-time PCR as described in the Materials and Methods. The results are presented as the fold induction of relative quantification by classification in ascending order. A positive fold change of 1 indicated 2-fold up-regulation, and a negative fold change of -1 indicated 2-fold down-regulation. A comparative analysis was performed between (A) high tumor stage *vs* low tumor stage, (B) high mSBR grades *vs* low mSBR grades, (C) HER2+ status *vs* HER2- status, and (D) recurrent patients *vs* non-recurrent patients. Statistical analysis was performed between groups using Student’s t or Kruskal Wallis tests (red bar: p < 0.05; black bars: p < 0.10).

The expression of selected genes was quantified by real-time quantitative PCR using Taqman low-density arrays (Applied Biosystems). The relative quantification (RQ) of each gene was determined in 32 tumor samples from 32 patients with breast carcinoma (S1 Table). Several groups of patients were defined according to tumor stage (tumor stage 2–3 *vs* tumor stage 1), histological grade (high mSBR grades *vs* low mSBR grades), HER2 status (HER2+ *vs* HER2-), and relapse occurrence (recurrent *vs* non-recurrent patients). The distribution of patients according to these clinicopathological criteria is presented in Table 1. A comparative analysis of gene expression based on the fold induction values was performed between different groups (Fig 1). Breast tumors were staged at diagnosis. The major tumor characteristic used to determine the stage was the tumor size. All patients have been diagnosed with early-stage invasive breast cancer. However, two groups were defined: a group of 7 patients with stage 1 and a group of 25 patients with stage 2 or 3. Only one patient was diagnosed with stage 3. The gene expression profile was randomly distributed, and no genes were significantly differently expressed between stages 2–3 and stage 1 (Fig 1A) (S2 Table). Patients were then divided into two groups based on the mSBR grading system modified by Le Doussal. This modified grading system was built from the nuclear pleomorphism and the mitotic index and retains five prognostic classes instead of three. Le Doussal *et al*. have demonstrated that mSRB grades 1, 2 and 3 have a lower risk for developing metastasis than mSRB grades 4 and 5. Seven patients were mSBR grade 4 or 5, and 25 patients were mSBR grade 1, 2 or 3. Overall, almost all genes were overexpressed in patients with high mSBR grades compared with low grades. The genes in the high-grade group were overexpressed by approximately 45% compared with the low-grade group. Interestingly, the *PTEN* gene was 6-fold down-expressed in high grades compared with low-grade tumors (p = 0.022). Insignificant differences (p < 0.10) were observed for *BRCA1*, *CDH1* and *NHERF1*, which are overexpressed in high-grade tumors, with increases of up to 2.2-, 1.6- and 1.5-fold, respectively (Fig 1B). Only 5 tumors were HER2+ (ICH+ and FISH+), and 27 tumors were HER2-. Overexpression of the majority of genes was observed in the group of patients with HER2+ breast cancer compared with the HER2- group. The genes in the HER+ group were overexpressed by an average of approximately 50% compared with HER- group. The *HER2* gene was overexpressed by more than 12-fold in the HER2+ group (p = 0.0007). The *NHERF1*, *PGK1* and *PHD2* genes were also significantly overexpressed (p = 0.028, p = 0.032 and p = 0.048, respectively). In addition, the *TGM2*, *CDH1*, *CTSD*, *FOXO3A* and *EDN1* genes were positively correlated with the HER2+ group (P < 0.10) (Fig 1C). A comparative analysis of gene expression profiles between recurrent and non-recurrent patients was also performed. With the exception of the *LEP* gene, all genes were overexpressed in the relapse group compared with the non-relapse group. The average gene overexpression in the relapse group was approximately 75%. Six genes was significantly overexpressed in the group of patients who relapsed: *EPO* (p = 0.013), *ETS1* (p = 0.022), *ENO1* (p = 0.003), *PGK1* (p = 0.021), *LDHA* (p = 0.011) and *TPI* (p = 0.048). In addition, *MET*, *VIM*, *CDH1*, *MMP2*, *VHL*, *FOXO3*, *VEGF*, *ABCG2* and *NDRG1* were associated with recurrent group (p < 0.10) (Fig 1D).

### Hierarchical clustering analysis of hypoxia-related gene expression

**Fig 2:**
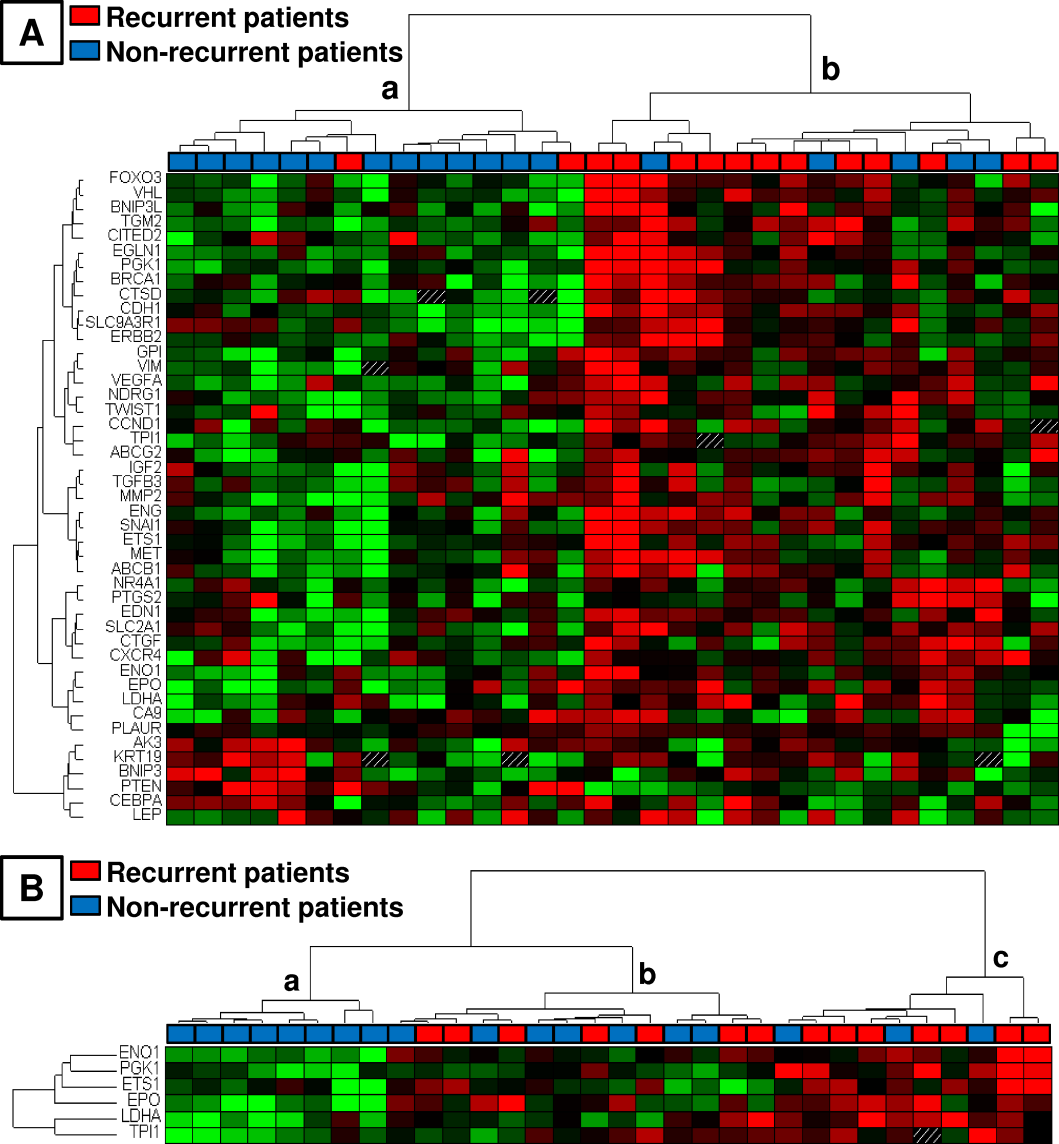
Profile of hypoxia-related gene expression in 32 tumors from patients with early-stage breast cancer. Data are presented in heat map format combined with hierarchical clustering using ΔCt values of gene expression. Each row represents a gene, and each column represents a patient. Gene expression is relative to the median of ΔCt values. Genes in red and green indicate expression above and below the median, respectively. (A) Hierarchical cluster analysis using all selected genes. (B) Hierarchical cluster analysis using the 6 differentially expressed genes with statistical significance between the recurrent group and non-recurrent group.

Data are presented in heat map format combined with hierarchical clustering, thus revealing the distribution of genes according to their expression in each tumor sample (Fig 2). Hierarchical clustering analysis of tumors based on the expression of all selected genes identified two main clusters of patients that were significantly associated with relapse occurrence (p = 0.008, Chi^2^ test). In cluster *a* and cluster *b*, 13% and 70% of patients relapsed, respectively (Fig 2A). The clustering based on the 6 significantly differentially expressed genes between the recurrent group and non-recurrent group (*EPO*, *ETS1*, *ENO1*, *PGK1*, *LDHA* and *TPI*) also significantly segregated patients who had relapsed: 0% of relapse in group *a*, 50% in *b* and 70% in *c* (p = 0.0095). For the comparison of groups *b* and *c* together with *a*, p = 0.03 (Fig 2B).

### Risk score of relapse

**Fig 3:**
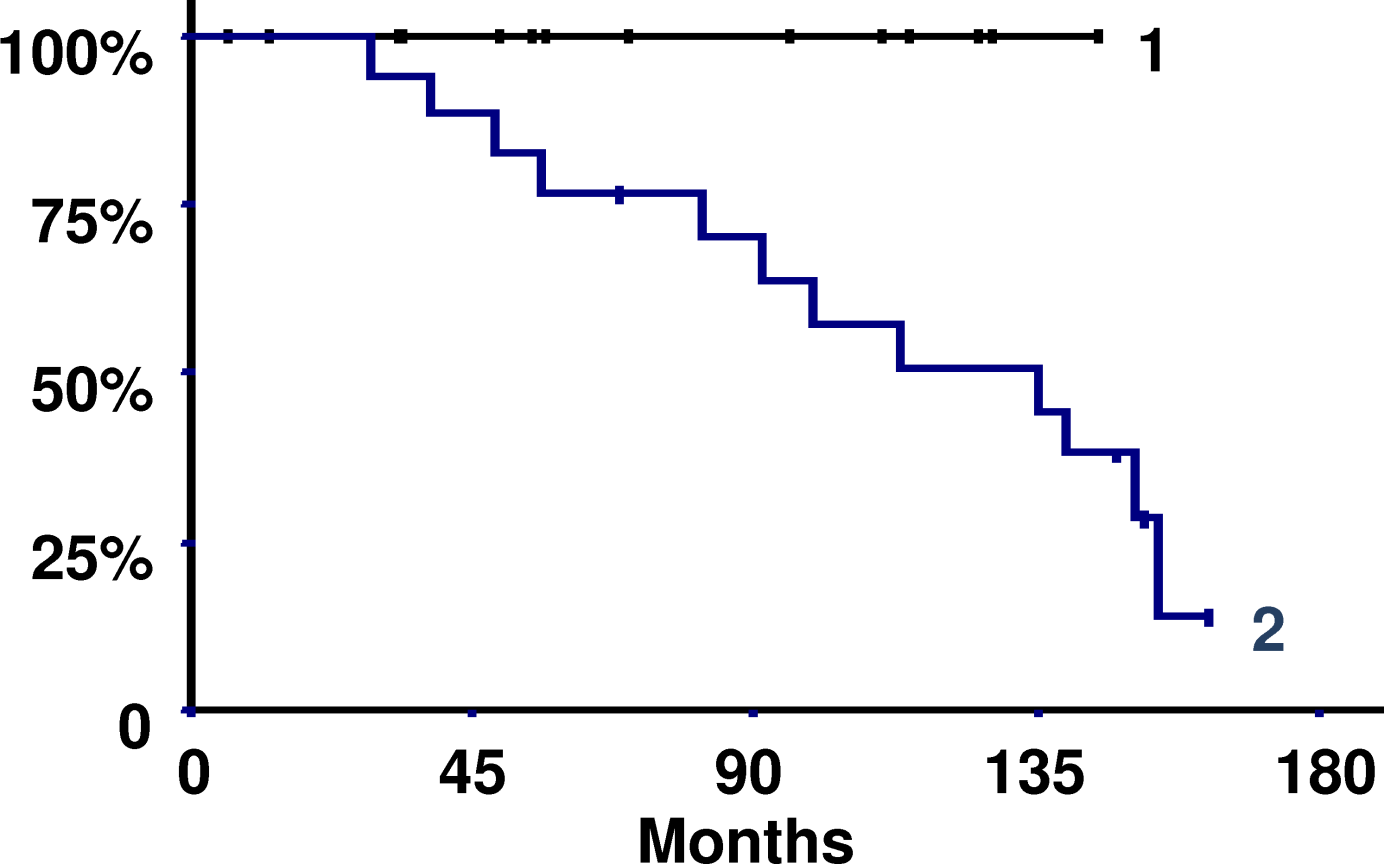
Kaplan-Meier relapse-free survival curves according to the risk score of relapse. Curve 1: 15 patients with score ≤ 2. Curve 2: 17 patients with score ≥ 3. The 14 recurrent patients were in curve 2 (p = 0.021, Mantel-Haenszel test).

**Fig 4:**
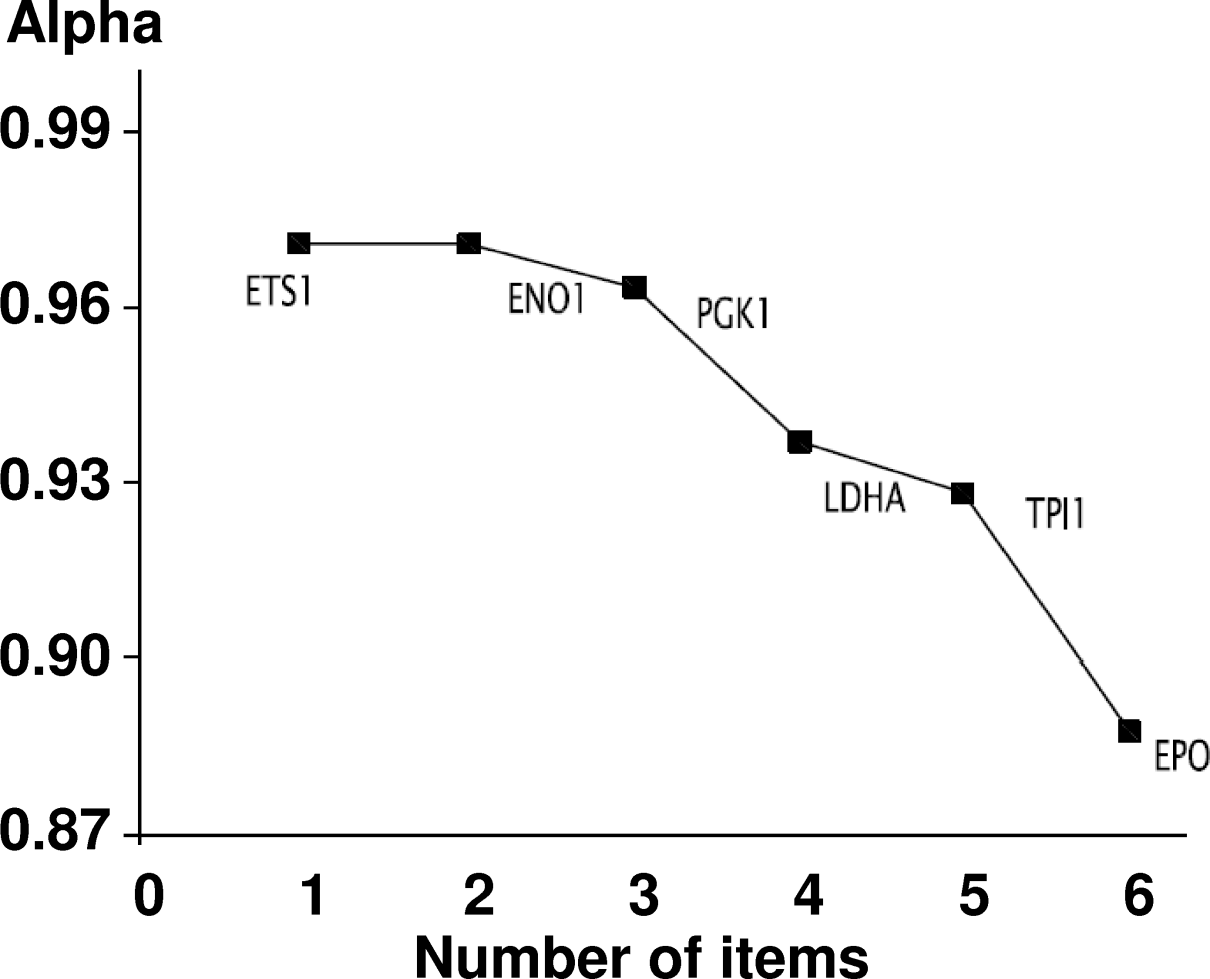
Analysis of the internal consistency of the 6 genes differentially expressed between recurrent and non-recurrent patients. Cronbach’s alpha coefficient was calculated to measure internal consistency (alpha = 0.90).

The comparison of gene expression between the relapse group and non-relapse group allowed the identification of six significantly differentially expressed genes: *EPO*, *ETS1*, *ENO1*, *PGK1*, *LDHA* and *TPI*. A basic algorithm was developed to classify patients according to a risk score of relapse. To define this score, the optimum level of each gene significantly expressed in the relapse group was determined by an iterative approach using the difference in relapse-free survival as the main criterion (Table 3). For each one of the six genes, a value of 1 was given if its expression was higher than the optimum thresholds presented in Table 3. The risk score was then calculated by summing the values attributed to each gene. Analysis of the Kaplan-Meier relapse-free survival curves using the Mantel-Haenszel test statistic permitted the definition of a threshold score of 2 (Fig 3). As shown in Fig 3, a threshold score equal to 2 yielded a significant difference between recurrent and non-recurrent patients (p = 0.021). The risk of relapse was multiplied by 1.384 if the score was ≥ 3, which indicated that the risk of relapse was increased by 40%. In the group with a score ≥ 3, the relapse rate was 19% after 5 years and 42% after 10 years; by contrast, the rate was 0% in the other group because no patient belonging to this group had relapsed. In addition, the statistical index of Cronbach's alpha [27] indicated that there was good consistency between all markers (alpha = 0.9) (Fig 4). In summary, the analysis of the expression values of *EPO*, *ETS1*, *ENO1*, *PGK1*, *LDHA* and *TPI* permitted the generation of a risk score of relapse in which a risk score of ≥ 3 indicates a short relapse time and a risk score ≤ 2 indicates a long relapse time.

**Table 3:** Optimum level of gene expression thresholds discriminating relapse-free survival.

*Gene*	Optima
*EPO*	7.10
*ETS1*	1.81
*ENO1*	1.00
*PGK1*	1.37
*LDHA*	1.20
*TPI*	1.14

## Discussion

A number of experimental and clinical studies have shown that adaptations of tumor cells to hypoxia are associated with malignant progression and development of resistance to both chemotherapy and radiotherapy [5, 28]. The expression of various hypoxic markers in breast cancer has been linked to a worse prognosis. In a clinical series of breast cancer patients, resistance to endocrine therapy combined with chemotherapy has been associated with overexpression of the HIF-1 alpha and CA9 proteins [29]. Cellular adaptations to hypoxia involve transcriptional modifications responsible for tumor aggressiveness and resistance to treatment [30]. Many approaches have sought to target the cellular response to hypoxia in human cancers [31, 32]. Inhibition of HIF-1 alpha activation appears to be the main approach and may improve the response to chemotherapy. Analysis of hypoxia-related gene expression may be useful in the development of novel therapeutic strategies for breast cancer. Several authors have identified different molecular signatures predicting the clinical outcome of cancer diseases; but these signatures differ greatly and share quite a few genes [21, 33, 34]. The aim of this study was to quantify the expression of well-known hypoxia-related genes in primary tumors from patients with early-stage breast cancer to assess their potential value as prognostic and predictive markers for cancer development and relapse occurrence. All patients included in this study received chemotherapy and endocrine therapy after initial surgery.

Multiple genes have been reported to be regulated by HIF complexes. These hypoxia regulated genes are involved in biological processes allowing tumor progression, such as cell proliferation and differentiation, survival, glucose metabolism, angiogenesis, migration, motility and drug resistance [12, 35]. A qualitative review of relevant literature related to tumor hypoxia and breast cancer enabled the selection of a set of candidate genes. Among these genes, we established a molecular signature composed of 45 genes involved in hypoxia signaling pathways and breast cancer progression. The expression of selected genes was quantified in a retrospective series including 32 tumor samples derived from patients with early-stage invasive ductal carcinoma without treatment at diagnosis. A comparison analysis of gene expression was performed according to clinicopathological features (stage, mSBR grade, HER2 status) and relapse occurrence.

Analysis of gene expression did not appear to be influenced by the clinical stage of tumors previously defined from the tumor node metastasis (TNM) classification. All patients were diagnosed with stage 1 or 2 breast cancer, with the exception of one patient with stage 3. The distribution of gene expression according to clinical stage showed no significant difference between stage 1 and stages 2 and 3. All tumors were less than 5 cm (T1 or T2) and were node-negative or 1 to 3 lymph-node positive (N0 or N1). These clinical criteria defined a relatively homogeneous group with no distant metastasis (M0). A hypoxic microenvironment has been consistently identified as a feature that promotes metastatic processes in breast cancer [36]. HIF factors regulate the transcription of several genes involved in different steps of the metastatic process, including angiogenesis, extracellular matrix modulation, cell migration and adhesion [37]. High proportions of hypoxic cells and increased levels of HIF-1 alpha protein in primary tumors of breast cancer patients indicate increased risk of metastasis and decreased overall survival rates [38]. Bos *et al*. revealed that high levels of HIF-1 alpha were significantly associated with overall survival in patients with negative lymph node status. However, no correlation was observed between levels of HIF-1 alpha expression and tumor size or lymph node status in a retrospective series of early-stage breast tumors [39]. In agreement with this finding, the expression of the hypoxia-regulated genes selected in this study was not associated with stage in this series of breast cancer patients.

In contrast to clinical stage, overexpression of almost all markers was observed in the group of patients with high-grade tumors. This group was also characterized by a significant decrease in *PTEN* gene expression. *PTEN* is a tumor suppressor that encodes a phosphatase involved in downregulation of the PI3K/AKT signaling pathway. The *PTEN* gene is frequently mutated or inactivated in multiple human cancers, including a large proportion of breast cancers. A number of clinical studies have demonstrated that loss or reduced expression of *PTEN* is involved in breast cancer progression, poor prognosis and resistance to treatment [40]. *PTEN* is also a negative regulator of HIF-1 alpha expression [10]. Some mutations or deletions of *PTEN* induce hyperactivation of the PI3K/AKT signaling pathway and activation of HIF complexes. In vitro studies of *PTEN* knockout in cancer cell lines have provided evidence for the role of *PTEN* in the stability and activity of the HIF-1 complex [10]. Tumor hypoxia and loss of *PTEN* function result in activation of HIF factors, followed by increased transcription of hypoxia-related genes and the development of a more aggressive breast cancer.

A similar analysis was performed in the group of patients with HER2+ breast cancer *vs* the group of HER2- patients. As expected, patients with HER2+ status harbored strong amplification of the *HER2* gene. In addition, significant differences in gene expression were observed for *NHERF1* (Na/H exchange regulatory factor), *PHD2* (prolyl-hydroxylase 2) and *PGK1* (phosphoglycerate kinase 1). The *NHERF1* gene encodes a protein capable of interacting with the HER2 receptor [41]. The mechanism of action of *NHERF1* in tumor cells has not been elucidated, but it has been reported that *NHERF1* plays an important role in cancer development. *NHERF1* overexpression is associated with high-grade tumors and increased expression of HIF-1 alpha protein in breast cancer [42]. Transcriptional activation of *NHERF1* by hypoxia has also been established in *in vitro* models, including several breast cancer cell lines [43]. The protein encoded by the *PHD2* gene is a dioxygenase that catalyzes the post-translational hydroxylation of HIF-1 alpha protein under normoxia. This enzyme plays a central role in the regulation and stability of HIF complexes. In vitro studies have demonstrated that levels of *PHD2* expression are increased in hypoxic conditions. The promoter of *PHD2* contains HRE elements, allowing the establishment of a positive feedback loop under hypoxia [44, 45]. In addition, increased levels of PHD2 protein have been correlated with relapse and tumor metastasis [46].

The *PGK1* gene was also significantly overexpressed in the group of patients with high mSBR grade as well as in the group of recurrent patients. Indeed, the comparative analysis of gene expression between recurrent patients and non-recurrent patients revealed overexpression of almost all genes. The *PGK1*, *LDHA*, *TPI*, *ENO1*, *EPO* and *ETS1* markers were significantly overexpressed in the relapse group compared with the non-relapse group. Among these 6 significantly differentially expressed genes, *PGK1*, *ENO1* (enolase), *LDHA* (lactate dehydrogenase) and *TPI* (triose phosphate isomerase) are directly involved in glucose metabolism and encode glycolytic enzymes. HIF factors have long been implicated in the regulation of genes involved in glucose metabolism in tumor cells [47]. These genes have HRE elements in their respective promoters and therefore bind HIF complexes [48]. In hypoxia, cancer cells redirect their aerobic metabolism to anaerobic metabolism by activating glycolysis, which becomes the main source of energy. Several other genes targeted by HIF factors are involved in multiple steps of glucose metabolism and are up-regulated under hypoxia. Overexpression of the TPI, PGK1 and ENO1 enzymes has been demonstrated in a series of breast tumors [49]. Expression of LDHA is increased in hypoxic tumor cells, leading to increased ATP production and cell proliferation. This enzyme catalyzes the conversion of pyruvate into lactate under hypoxia. The lactate is absorbed by non-hypoxic tumor cells for use as a respiratory substrate for promoting angiogenesis and metastasis [50]. In several breast cancer cell lines, inactivation of LDHA inhibits cell proliferation and induces apoptosis [51]. The *EPO* gene encodes erythropoietin, which is a specific stimulator of erythropoiesis [52]. The HIF-1 factor was discovered by the identification of HRE elements in the promoter of *EPO* [53]. Regulation of *EPO* by HIF complexes under hypoxic conditions is well documented [54]. EPO is a potent inhibitor of apoptosis caused by ischemia and hypoxia [55]. In erythrocytes, binding of EPO to its receptor (EPOR) results in the activation of multiple signaling pathways responsible for cell proliferation and differentiation [56, 57]. EPO and its receptor are also expressed in other cell types, including endothelial cells and mammary epithelial cells [58]. High mRNA and protein levels of EPO and EPOR have been reported in several cancer cell lines. In vitro studies in breast cancer cell lines have demonstrated that autocrine/paracrine production of EPO and EPOR under hypoxia contributes to cell survival and proliferation. Other authors have shown that the EPO/EPOR axis plays an important role in the regulation of the migration and invasion of breast cancer cells [59]. The *ETS1* gene is a proto-oncogene encoding a transcription factor involved in the proliferation of normal breast epithelial cells. This gene is also involved in tumor progression in breast cancers and contributes to aggressive tumor phenotypes by activating the transcription of genes involved in angiogenesis, extracellular matrix remodeling, cell adhesion and invasion [60]. In addition, HRE elements have been identified in the promoter of *ETS1*, suggesting transcriptional activation under hypoxic conditions [60]. Span *et al*. demonstrated that increased expression of *ETS1* was associated with increased risk of recurrence in a series of invasive breast cancers. In agreement with these previous studies, overexpression of this 6-gene set appears to be involved in tumor progression contributing to the occurrence of relapse.

Overall, the expression of the 45-gene set was associated with aggressive tumors characterized by high grade, HER2+ status and increased recurrence risk. This gene signature reflects the impact of the hypoxic microenvironment on cancer cells. Our findings provide further evidence that hypoxia-related genes are involved in the clinical outcome of breast cancer by activating hypoxia signaling pathways. Although this study is based on a limited number of patients, assessment of hypoxia-related gene expression in breast cancer could have potential prognostic value. In particular, quantification of the expression of *EPO*, *ETS1*, *PGK1*, *TPI*, *LDHA* and *ENO1* in a primary tumor sample provides information on the risk of recurrence for patients with early-stage invasive breast cancer. The calculation of a score from the expression of this 6-gene set permitted the classification of patients with a low or high risk of relapse. A primary breast tumor with a risk score ≥ 3 has a high risk of recurrence, and a tumor with a risk score ≤ 2 has a low risk of recurrence. Furthermore, hierarchical clustering analysis of gene expression identified two main groups of patients significantly associated with relapse occurrence.

In summary, we have defined a molecular signature specific to hypoxia responses in breast cancer. This gene signature was associated with tumor aggressiveness and the risk of recurrence. The expression of the 6-gene set allowed the calculation of a relapse risk score. In addition to existing clinicopathological parameters, we showed that the assessment of hypoxia-related gene expression using simple real-time PCR assays in frozen breast tumor samples could improve the prediction of recurrence risk in breast cancer. Although this study has some limitations, such as its retrospective nature and the limited number of patients, our results provide additional clinical evidence that hypoxia-related gene expression has prognostic potential. Of course, it will be necessary to validate the clinical relevance of the risk score based on these 6 genes in independent studies including larger prospective patient cohorts. In addition, this risk score provides a prediction of relapse likelihood regardless of treatment type. Thus, it will be interesting to assess the potential value of the risk score of relapse following specific therapies.

## Supporting information

S1 Table Clinical and histopathological criteria of patients, and relative quantification of genes. (DOCX)

S2 Table Average relative quantification, standard deviation and fold induction for each gene expression in a group of patients relative to the corresponding control group. (DOCX)
