# Supplementary material for: Quantification of hypoxia-related gene expression as a potential approach for clinical outcome prediction in breast cancer
Source: PLoS One. 2017 Apr 21;12(4):e0175960. doi: 10.1371/journal.pone.0175960 (PMC5400273; doi:10.1371/journal.pone.0175960)
Supplement: S1 Table — (DOCX) [file pone.0175960.s001.docx]

****S1 Table. Clinical and histopathological criteria of patients, and relative quantification of genes.****

| Patients ID | 1 | 2 | 3 | 4 | 5 | 6 | 7 | 8 | 9 | 10 | 11 | 12 | 13 | 14 | 15 | 16 | 17 | 18 | 19 | 20 | 21 | 22 | 23 | 24 | 25 | 26 | 27 | 28 | 29 | 30 | 31 | 32 |
| --- | --- | --- | --- | --- | --- | --- | --- | --- | --- | --- | --- | --- | --- | --- | --- | --- | --- | --- | --- | --- | --- | --- | --- | --- | --- | --- | --- | --- | --- | --- | --- | --- |
| Tumor stage | 2 | 1 | 1 | 3 | 2 | 2 | 2 | 2 | 2 | 2 | 2 | 2 | 2 | 2 | 2 | 2 | 2 | 2 | 2 | 2 | 1 | 1 | 2 | 1 | 2 | 1 | 2 | 2 | 2 | 2 | 2 | 1 |
| mSBR grade | 4 | 2 | 3 | 3 | 2 | 3 | 2 | 4 | 3 | 3 | 4 | 5 | 2 | 4 | 3 | 4 | 2 | 3 | 2 | 3 | 3 | 1 | 2 | 3 | 2 | 3 | 4 | 3 | 2 | 3 | 3 | 1 |
| HER2 statut | 0 | 0 | 0 | 0 | 0 | 0 | 0 | 1+ | 3+ | 1+ | 3+ | 0 | 0 | 1+ | 0 | 0 | 1+ | 0 | 0 | 3+ | 0 | 0 | 0 | 0 | 0 | 0 | 3+ | 0 | 0 | 0 | 3+ | 0 |
| Relapse | n | n | y | y | y | n | y | y | n | y | y | n | n | n | y | n | n | n | n | n | y | n | n | n | n | y | y | n | n | n | y | n |
| Genes | Relative quantification | | | | | | | | | | | | | | | | | | | | | | | | | | | | | | | |
| ABCB1 | 8,4 | 2,5 | 6,6 | 7,2 | 5,8 | 0,8 | 1,5 | 17,1 | 7,8 | 1,9 | 0,9 | 2,5 | 2,3 | 1,9 | 1,3 | 4,0 | 2,1 | 0,9 | 3,8 | 1,9 | 18,9 | 1,8 | 14,5 | 1,9 | 2,8 | 0,9 | 10,5 | 2,3 | 4,0 | 3,4 | 2,8 | 1,0 |
| ABCG2 | 0,8 | 1,1 | 2,9 | 1,5 | 1,4 | 0,7 | 0,8 | 4,6 | 1,6 | 3,2 | 1,0 | 0,4 | 1,1 | 2,6 | 1,3 | 1,2 | 1,6 | 0,5 | 1,6 | 0,3 | 4,0 | 1,0 | 3,0 | 0,6 | 1,3 | 0,7 | 1,3 | 0,8 | 1,9 | 0,2 | 1,5 | 1,0 |
| AK3 | 0,3 | 0,7 | 1,0 | 1,3 | 0,8 | 0,5 | 0,8 | 3,2 | 1,3 | 0,5 | 0,2 | 0,3 | 0,6 | 0,9 | 0,7 | 1,4 | 1,0 | 1,8 | 1,4 | 1,5 | 1,6 | 2,3 | 1,6 | 0,8 | 0,6 | 1,2 | 0,6 | 0,7 | 1,1 | 1,0 | 0,8 | 1,0 |
| BNIP3 | 0,8 | 1,5 | 0,7 | 1,9 | 1,3 | 0,9 | 1,1 | 2,7 | 1,2 | 1,0 | 1,1 | 0,8 | 0,5 | 1,7 | 1,4 | 1,8 | 0,6 | 2,0 | 1,1 | 0,9 | 0,8 | 2,2 | 0,6 | 2,0 | 1,0 | 1,0 | 1,4 | 0,8 | 0,9 | 0,7 | 1,7 | 1,0 |
| BNIP3L | 1,3 | 1,7 | 1,6 | 1,2 | 0,8 | 1,2 | 0,8 | 9,1 | 4,6 | 0,4 | 0,9 | 0,5 | 1,0 | 0,9 | 0,5 | 0,6 | 1,4 | 0,5 | 3,2 | 0,6 | 6,4 | 1,3 | 1,2 | 1,1 | 1,0 | 0,6 | 1,2 | 0,5 | 0,8 | 0,4 | 1,4 | 1,0 |
| BRCA1 | 1,4 | 1,4 | 1,0 | 1,5 | 1,7 | 0,6 | 0,7 | 13,0 | 5,0 | 1,1 | 1,5 | 0,8 | 0,4 | 4,1 | 1,1 | 0,6 | 1,2 | 0,5 | 2,1 | 1,3 | 4,0 | 0,9 | 0,3 | 1,2 | 0,3 | 0,3 | 2,6 | 1,4 | 2,1 | 0,5 | 0,6 | 1,0 |
| CA9 | 2,0 | 99,0 | 0,5 | 1,2 | - | 1,9 | 50,3 | 116,2 | 146,0 | - | 0,2 | 4,8 | 0,8 | 0,5 | - | - | 3,5 | - | - | 4,4 | 205,5 | 17,5 | 1,1 | - | 16,8 | 52,0 | 0,8 | 1,9 | 6,3 | 157,2 | 43,3 | 1,0 |
| CCND1 | 0,5 | 0,8 | 1,1 | 1,9 | 1,3 | 0,2 | 0,4 | 9,5 | 4,2 | 2,1 | 0,3 | 0,5 | 1,8 | 2,0 | 0,8 | 0,6 | 0,4 | 1,0 | 0,4 | 0,2 | 2,2 | 0,4 | 0,3 | 1,3 | 0,4 | 0,2 | 1,1 | 0,3 | 0,8 | 0,1 | 0,7 | 1,0 |
| CDH1 | 2,1 | 1,6 | 2,2 | 2,8 | 1,6 | 1,4 | 0,8 | 29,7 | 18,3 | 4,3 | 5,0 | 0,7 | 1,1 | 4,3 | 1,4 | 1,6 | 0,9 | 1,9 | 4,3 | 0,8 | 6,8 | 1,2 | 0,6 | 2,3 | 0,9 | 0,3 | 4,9 | 0,3 | 3,5 | 0,8 | 3,6 | 1,0 |
| CEBPA | 1,1 | 1,1 | 1,0 | 2,2 | 1,3 | 1,1 | 0,6 | 4,8 | 1,2 | 0,7 | 0,8 | 0,8 | 1,6 | 1,3 | 0,3 | 1,4 | 1,0 | 1,9 | 1,1 | 1,4 | 1,7 | 1,4 | 1,4 | 1,5 | 0,7 | 1,3 | 1,8 | 0,8 | 0,7 | 0,8 | 0,7 | 1,0 |
| CITED2 | 1,0 | 1,2 | 1,3 | 1,3 | 0,7 | 0,8 | 0,7 | 3,8 | 3,3 | 0,6 | 1,0 | 0,6 | 1,1 | 0,7 | 0,8 | 0,3 | 2,9 | 2,2 | 1,6 | 1,0 | 4,2 | 1,4 | 0,9 | 0,8 | 0,8 | 0,5 | 1,3 | 0,7 | 2,5 | 0,5 | 1,9 | 1,0 |
| CTGF | 0,8 | 6,2 | 3,1 | 1,5 | 0,8 | 0,9 | 4,5 | 21,7 | 2,5 | 2,6 | 2,8 | 1,2 | 4,4 | 2,8 | 0,7 | 1,6 | 2,1 | 0,8 | 4,0 | 2,8 | 6,1 | 0,9 | 2,3 | 1,4 | 1,6 | 1,6 | 1,2 | 1,4 | 3,0 | 2,4 | 1,6 | 1,0 |
| CTSD | 1,2 | 0,7 | 0,4 | 0,7 | 0,5 | 0,1 | 0,2 | 2,7 | 3,3 | 0,3 | 0,9 | 0,2 | 0,4 | 0,3 | 0,9 | 0,3 | 0,1 | 0,2 | 0,5 | 0,3 | 1,0 | 0,6 | 0,1 | 0,4 | 0,4 | 0,1 | 3,8 | 0,2 | 0,2 | 0,2 | 0,3 | 1,0 |
| CXCR4 | 5,2 | 3,7 | 3,0 | 2,7 | 2,4 | 2,2 | 4,4 | 9,5 | 3,3 | 2,5 | 2,0 | 1,6 | 4,0 | 3,2 | 0,8 | 1,0 | 3,7 | 1,2 | 2,3 | 4,4 | 3,9 | 2,7 | 1,9 | 2,8 | 2,1 | 1,2 | 2,7 | 2,5 | 3,3 | 1,6 | 2,0 | 1,0 |
| EDN1 | 4,3 | 4,5 | 6,6 | 5,1 | 6,5 | 0,2 | 10,3 | 38,2 | 11,0 | 2,6 | 7,6 | 4,5 | 29,4 | 2,3 | 1,3 | 3,9 | 3,3 | 0,6 | 7,1 | 6,1 | 29,0 | 1,8 | 2,8 | 5,5 | 8,2 | 2,2 | 10,4 | 6,1 | 2,9 | 6,0 | 4,2 | 1,0 |
| EGLN1 | 1,5 | 3,1 | 2,5 | 2,7 | 1,9 | 1,3 | 1,2 | 22,0 | 10,5 | 2,2 | 3,9 | 1,6 | 1,3 | 1,3 | 1,2 | 1,1 | 2,4 | 1,3 | 4,3 | 1,4 | 11,5 | 2,6 | 1,4 | 1,2 | 1,0 | 0,7 | 5,4 | 2,6 | 2,0 | 1,3 | 2,5 | 1,0 |
| ENG | 0,8 | 0,9 | 1,1 | 2,0 | 0,9 | 0,2 | 0,6 | 6,2 | 2,5 | 1,1 | 1,5 | 0,4 | 1,1 | 0,8 | 0,5 | 0,8 | 0,9 | 0,7 | 1,5 | 0,5 | 3,6 | 0,6 | 1,6 | 0,6 | 1,0 | 0,5 | 2,5 | 0,7 | 1,4 | 0,9 | 0,6 | 1,0 |
| ENO1 | 1,1 | 2,2 | 1,2 | 1,2 | 1,4 | 0,9 | 1,6 | 9,0 | 1,7 | 1,2 | 1,7 | 1,2 | 1,3 | 1,4 | 1,7 | 0,8 | 0,8 | 0,4 | 1,2 | 0,7 | 6,2 | 1,3 | 0,9 | 0,7 | 1,1 | 1,0 | 1,4 | 0,8 | 0,9 | 1,7 | 1,5 | 1,0 |
| EPO | 3,0 | 41,9 | 42,6 | 1,8 | 12,1 | - | 71,7 | 87,2 | 20,9 | 1,9 | 132,2 | 50,9 | 2,5 | 5,6 | 15,2 | 0,1 | 1,1 | - | 7,1 | - | 19,9 | 1,7 | 1,1 | - | 5,2 | 184,3 | 14,0 | 0,7 | 42,5 | 22,4 | 1,4 | 1,0 |
| ERBB2 | 1,0 | 1,1 | 0,7 | 2,7 | 1,4 | 0,6 | 0,7 | 9,6 | 45,8 | 4,2 | 36,3 | 0,4 | 0,7 | 2,3 | 0,6 | 0,9 | 3,0 | 1,2 | 2,5 | 1,9 | 5,2 | 0,8 | 0,6 | 0,9 | 0,6 | 0,2 | 31,1 | 1,3 | 2,8 | 0,2 | 1,6 | 1,0 |
| ETS1 | 2,6 | 2,3 | 2,9 | 2,6 | 2,2 | 0,4 | 1,4 | 10,8 | 1,8 | 2,2 | 1,4 | 1,3 | 1,6 | 1,8 | 0,6 | 1,3 | 1,1 | 0,6 | 1,3 | 1,0 | 9,1 | 0,7 | 1,8 | 1,4 | 1,2 | 1,4 | 2,9 | 1,3 | 1,0 | 1,6 | 0,9 | 1,0 |
| FOXO3 | 1,4 | 1,1 | 1,6 | 1,1 | 0,8 | 0,3 | 0,8 | 6,9 | 3,4 | 0,7 | 1,1 | 0,7 | 0,4 | 0,7 | 0,4 | 0,6 | 0,9 | 0,3 | 1,6 | 0,6 | 3,5 | 0,6 | 0,7 | 0,6 | 0,5 | 0,4 | 1,2 | 0,7 | 1,0 | 0,4 | 1,2 | 1,0 |
| GPI | 1,1 | 1,7 | 1,5 | 1,3 | 0,8 | 1,0 | 0,6 | 7,8 | 1,8 | 1,2 | 1,3 | 0,7 | 0,9 | 1,3 | 0,5 | 0,8 | 1,1 | 0,5 | 1,5 | 0,6 | 2,8 | 0,9 | 0,6 | 0,8 | 1,3 | 1,7 | 1,8 | 0,9 | 1,2 | 0,9 | 1,4 | 1,0 |
| IGF2 | 0,4 | 3,6 | 11,3 | 2,5 | 1,3 | 0,2 | 1,6 | 8,2 | 1,4 | 2,2 | 0,8 | 0,9 | 1,7 | 2,6 | 0,3 | 4,7 | 3,7 | 0,8 | 1,8 | 1,0 | 11,6 | 0,9 | 5,2 | 1,6 | 1,4 | 1,3 | 6,5 | 2,2 | 2,5 | 0,8 | 2,4 | 1,0 |
| KRT19 | 0,6 | 1,9 | 1,0 | 2,8 | 2,4 | 1,5 | 2,0 | 6,4 | 5,1 | 2,8 | 0,9 | 0,9 | 1,3 | 4,8 | 3,8 | 3,2 | 1,5 | 4,5 | 2,7 | 5,8 | 6,2 | 5,2 | 1,4 | 2,7 | 1,1 | 1,1 | 2,5 | 2,1 | 4,3 | 0,9 | 3,1 | 1,0 |
| LDHA | 0,9 | 1,7 | 1,3 | 2,3 | 1,0 | 1,4 | 2,3 | 3,7 | 1,9 | 0,7 | 1,8 | 0,9 | 0,9 | 1,3 | 1,5 | 0,5 | 0,6 | 0,6 | 0,7 | 0,7 | 1,6 | 1,2 | 0,6 | 0,8 | 1,0 | 0,8 | 1,2 | 0,5 | 0,9 | 1,1 | 2,1 | 1,0 |
| LEP | 0,2 | 0,2 | 0,4 | 3,6 | 1,2 | 0,4 | - | 2,7 | 8,1 | 0,2 | 0,0 | 0,1 | 0,9 | 3,8 | 0,5 | 0,1 | 1,2 | 0,2 | 0,1 | 0,2 | 0,6 | 11,5 | 5,6 | 0,1 | 1,5 |  | 3,6 | 0,1 | 3,6 | 0,8 | 0,1 | 1,0 |
| MET | 6,6 | 5,8 | 12,3 | 6,8 | 5,7 | 0,4 | 0,9 | 26,9 | 23,2 | 2,8 | 16,5 | 1,1 | 2,5 | 2,1 | 1,1 | 3,6 | 3,9 | 0,5 | 2,0 | 1,0 | 60,0 | 1,7 | 8,2 | 3,2 | 2,0 | 1,1 | 21,4 | 2,2 | 2,6 | 6,2 | 2,7 | 1,0 |
| MMP2 | 2,1 | 10,8 | 15,5 | 8,5 | 2,8 | 1,7 | 8,0 | 19,3 | 5,6 | 2,7 | 9,7 | 5,2 | 5,2 | 2,9 | 1,1 | 6,5 | 3,5 | 1,5 | 4,8 | 3,0 | 28,3 | 2,6 | 15,8 | 4,2 | 3,5 | 8,3 | 6,5 | 10,4 | 3,1 | 7,9 | 7,6 | 1,0 |
| NDRG1 | 1,7 | 7,2 | 2,9 | 4,2 | 2,5 | 1,7 | 3,9 | 14,0 | 15,2 | 2,8 | 4,1 | 1,3 | 2,0 | 9,7 | 0,6 | 3,1 | 2,3 | 2,0 | 2,8 | 1,2 | 9,3 | 1,5 | 2,4 | 2,5 | 1,6 | 4,5 | 2,9 | 1,6 | 6,8 | 4,2 | 2,3 | 1,0 |
| NR4A1 | 1,8 | 9,9 | 2,8 | 4,0 | 4,0 | 2,1 | 18,4 | 12,2 | 7,6 | 1,4 | 1,6 | 1,5 | 26,0 | 8,4 | 3,5 | 2,0 | 3,3 | 2,8 | 5,4 | 6,5 | 5,3 | 2,1 | 0,8 | 4,1 | 3,4 | 1,8 | 3,5 | 2,2 | 3,4 | 2,3 | 1,7 | 1,0 |
| PGK1 | 1,3 | 2,6 | 1,9 | 1,7 | 1,5 | 0,9 | 1,6 | 14,8 | 5,1 | 1,4 | 4,0 | 1,3 | 1,7 | 3,0 | 1,4 | 1,0 | 1,6 | 1,6 | 2,5 | 1,4 | 5,6 | 1,2 | 0,8 | 0,9 | 1,7 | 1,4 | 3,8 | 1,4 | 1,6 | 1,4 | 1,7 | 1,0 |
| PLAUR |  | 7,5 | 4,4 | 3,2 | 2,0 | 0,8 | 5,2 | 32,6 | 5,4 | - | 3,6 | 1,5 | 4,2 | 2,2 | 1,2 | 1,3 | 1,4 | 0,6 | 4,6 | 2,7 | 16,1 | 1,1 | 1,8 | 3,2 | 3,7 | 3,0 | 3,9 | 2,6 | 1,7 | 3,8 | 2,4 | 1,0 |
| PTEN | 0,2 | 0,2 | 0,4 | 0,5 | 0,5 | 1,8 | 0,5 | 0,2 | 0,1 | 0,3 | 0,3 | 0,1 | 0,5 | 0,3 | 16,0 | 1,0 | 0,9 | 5,8 | 0,3 | 5,9 | 0,2 | 3,1 | 0,6 | 0,6 | 0,5 | 10,0 | 0,2 | 0,4 | 0,3 | 7,2 | 0,2 | 1,0 |
| COX2 | 6,4 | 27,9 | 6,4 | 9,3 | 6,9 | 2,4 | 51,0 | 13,2 | 6,7 | 0,9 | 3,6 | 1,2 | 18,7 | 25,2 | 9,1 | 4,4 | 6,6 | 53,5 | 3,3 | 9,4 | 9,3 | 5,2 | 8,0 | 4,9 | 6,6 | - | 4,8 | 2,0 | 6,6 | 7,7 | 2,1 | 1,0 |
| GLUT1 | 1,4 | 2,9 | 1,5 | 2,5 | 0,9 | 0,4 | 1,5 | 4,7 | 5,4 | 2,7 | 1,5 | 0,9 | 2,2 | 2,6 | 0,6 | 1,7 | 1,1 | 1,0 | 3,3 | 1,6 | 5,5 | 0,6 | 0,5 | 2,2 | 1,1 | 0,7 | 2,1 | 2,1 | 2,3 | 2,0 | 1,3 | 1,0 |
| NHERF1 | 1,0 | 1,4 | 1,1 | 1,7 | 1,1 | 1,1 | 0,7 | 4,6 | 4,5 | 1,9 | 4,9 | 0,3 | 1,9 | 7,7 | 2,4 | 2,3 | 0,8 | 2,1 | 1,6 | 1,8 | 3,1 | 0,8 | 0,4 | 2,3 | 0,7 | 0,3 | 4,2 | 0,4 | 1,7 | 0,5 | 1,4 | 1,0 |
| SNAI1 | 1,3 | 2,4 | 5,3 | 3,1 | 1,1 | 0,5 | 2,3 | 21,8 | 5,4 | 2,4 | 3,4 | 0,8 | 2,6 | 1,5 | 0,4 | 2,1 | 1,7 | 0,6 | 2,9 | 1,4 | 9,8 | 0,9 | 2,6 | 1,7 | 1,8 | 1,0 | 3,0 | 1,6 | 3,8 | 1,1 | 1,5 | 1,0 |
| TGFB3 | 0,8 | 2,6 | 4,7 | 2,3 | 0,9 | 0,8 | 1,2 | 5,5 | 2,3 | 1,2 | 1,1 | 1,0 | 1,3 | 1,2 | 0,8 | 1,9 | 2,2 | 1,1 | 1,1 | 1,8 | 10,0 | 1,2 | 2,7 | 1,1 | 2,0 | 1,8 | 3,4 | 1,7 | 1,2 | 0,9 | 1,5 | 1,0 |
| TGM2 | 3,6 | 6,6 | 2,3 | 2,1 | 5,3 | 0,8 | 1,2 | 9,3 | 21,1 | 1,0 | 1,4 | 1,2 | 2,5 | 0,7 | 0,4 | 0,9 | 1,5 | 0,3 | 3,8 | 1,0 | 5,5 | 1,1 | 2,1 | 1,1 | 0,9 | 1,0 | 5,9 | 1,1 | 6,1 | 3,9 | 13,2 | 1,0 |
| TPI1 | 0,8 | 1,1 | 1,3 | 0,8 | 0,7 | 1,0 | 0,8 | 2,9 | 1,5 | 1,3 | 1,5 | 0,6 | 1,0 | 3,0 | 1,0 | 0,5 | 0,5 | 0,7 | 1,0 | 0,5 | 1,4 | 0,9 | 0,7 | 0,7 | 0,8 | 0,7 | 1,1 | 0,5 | 1,0 | 0,8 | 1,1 | 1,0 |
| TWIST1 | 2,4 | 7,2 | 12,9 | 7,4 | 2,0 | 2,1 | 3,4 | 31,0 | 5,4 | 3,0 | 5,5 | 2,5 | 2,8 | 24,1 | 1,0 | 3,7 | 3,8 | 26,4 | 1,7 | 3,1 | 15,4 | 2,7 | 8,1 | 2,5 | 2,9 | 4,2 | 4,1 | 5,1 | 32,8 | 5,1 | 5,4 | 1,0 |
| VEGFA | 0,4 | 1,3 | 0,5 | 1,1 | 0,4 | 0,4 | 0,7 | 2,7 | 1,6 | 1,3 | 0,4 | 0,3 | 0,4 | 0,6 | 0,5 | 0,4 | 0,4 | 0,2 | 1,0 | 0,4 | 2,9 | 0,3 | 0,2 | 0,3 | 0,5 | 0,7 | 0,6 | 0,3 | 0,9 | 0,6 | 0,4 | 1,0 |
| VHL | 1,0 | 1,3 | 1,5 | 1,7 | 1,0 | 0,4 | 0,7 | 5,5 | 1,9 | 1,1 | 0,9 | 0,8 | 0,8 | 0,9 | 0,7 | 0,7 | 1,0 | 0,6 | 1,2 | 0,6 | 4,4 | 0,7 | 0,6 | 0,7 | 0,8 | 0,4 | 1,2 | 0,7 | 1,1 | 0,6 | 0,9 | 1,0 |
| VIM | 1,3 | 3,1 | 2,6 | 2,2 | 1,2 | 0,8 | 1,7 | 14,8 | 2,2 | 2,0 | 1,8 | 0,8 | 1,5 | 2,3 | 0,5 | 1,2 | 1,9 | 0,6 | 1,4 | 1,4 | 10,3 | 1,0 | 2,9 | 1,2 | 1,8 | 2,1 | 2,4 | 1,6 | 1,9 | 1,4 | 1,7 | 1,0 |
