# Supplementary material for: Quantification of hypoxia-related gene expression as a potential approach for clinical outcome prediction in breast cancer
Source: PLoS One. 2017 Apr 21;12(4):e0175960. doi: 10.1371/journal.pone.0175960 (PMC5400273; doi:10.1371/journal.pone.0175960)
Supplement: S2 Table — (DOCX) [file pone.0175960.s002.docx]

****S2 Table. Average relative quantification, standard deviation and**** fold induction for each gene expression in a group of patients relative to the corresponding control group.

| Genes | High tumor stage (HS) | | Low Tumor stage (LS) | | Fold Induction HS/LS | High mSBR grades (HG) | | Low mSBR grades (LG) | | Fold Induction HG/LG | HER2+ (H+) | | HER- (H-) | | Fold Induction H+/H- | Recurrent patients (R) | | Non-recurrent patients (NR) | | Fold Induction R/NR |
| --- | --- | --- | --- | --- | --- | --- | --- | --- | --- | --- | --- | --- | --- | --- | --- | --- | --- | --- | --- | --- |
|  | Mean | SD | Mean | SD |  | Mean | SD | Mean | SD |  | Mean | SD | Mean | SD |  | Mean | SD | Mean | SD |  |
| ABCB1 | 4,49 | 4,27 | 4,81 | 6,52 | -0,07 | 6,46 | 5,87 | 4,03 | 4,35 | 0,60 | 4,76 | 4,14 | 4,52 | 4,90 | 0,05 | 6,25 | 5,84 | 3,53 | 3,83 | 0,78 |
| ABCG2 | 1,45 | 1,00 | 1,62 | 1,32 | -0,12 | 1,67 | 1,33 | 1,43 | 0,95 | 0,17 | 1,14 | 0,52 | 1,55 | 1,12 | -0,35 | 1,90 | 1,26 | 1,16 | 0,76 | 0,74 |
| AK3 | 1,00 | 0,63 | 1,23 | 0,55 | -0,23 | 0,99 | 0,98 | 1,06 | 0,45 | -0,08 | 0,87 | 0,52 | 1,08 | 0,63 | -0,25 | 1,03 | 0,74 | 1,04 | 0,51 | 0,02 |
| BNIP3 | 1,19 | 0,52 | 1,35 | 0,61 | -0,13 | 1,38 | 0,69 | 1,16 | 0,48 | 0,19 | 1,25 | 0,29 | 1,22 | 0,57 | 0,02 | 1,29 | 0,53 | 1,16 | 0,56 | 0,16 |
| BNIP3L | 1,43 | 1,84 | 1,95 | 2,02 | -0,37 | 1,98 | 2,89 | 1,39 | 1,39 | 0,42 | 1,71 | 1,63 | 1,51 | 1,93 | 0,13 | 2,09 | 2,56 | 1,23 | 1,40 | 0,68 |
| BRCA1 | 1,86 | 2,59 | 1,39 | 1,20 | 0,34 | 3,16 | 4,14 | 1,29 | 1,10 | 1,45 | 2,20 | 1,70 | 1,68 | 2,46 | 0,31 | 2,34 | 3,22 | 1,35 | 1,40 | 0,80 |
| CA9 | 29,43 | 51,57 | 62,58 | 79,45 | -1,13 | 18,28 | 43,22 | 42,64 | 62,94 | -1,33 | 38,91 | 62,51 | 37,00 | 60,18 | 0,05 | 47,21 | 67,16 | 28,68 | 50,54 | 0,80 |
| CCND1 | 1,26 | 1,94 | 0,98 | 0,64 | 0,29 | 1,86 | 3,13 | 0,96 | 0,92 | 0,93 | 1,28 | 1,68 | 1,19 | 1,78 | 0,08 | 1,60 | 2,37 | 0,85 | 0,83 | 1,11 |
| CDH1 | 3,90 | 6,44 | 2,21 | 2,14 | 0,77 | 6,15 | 9,67 | 2,59 | 3,62 | 1,38 | 6,53 | 6,78 | 2,98 | 5,55 | 1,19 | 4,99 | 7,34 | 2,48 | 4,43 | 1,13 |
| CEBPA | 1,27 | 0,87 | 1,28 | 0,27 | -0,01 | 1,63 | 1,34 | 1,15 | 0,45 | 0,42 | 1,17 | 0,45 | 1,29 | 0,83 | -0,10 | 1,39 | 1,12 | 1,17 | 0,35 | 0,23 |
| CITED2 | 1,32 | 0,93 | 1,50 | 1,23 | -0,13 | 1,44 | 1,24 | 1,40 | 0,95 | 0,03 | 1,69 | 0,95 | 1,30 | 1,00 | 0,30 | 1,49 | 1,14 | 1,27 | 0,87 | 0,20 |
| COX2 | 10,78 | 13,61 | 9,09 | 9,58 | 0,19 | 8,17 | 7,70 | 11,05 | 13,93 | -0,35 | 5,31 | 2,85 | 11,44 | 13,75 | -1,16 | 9,70 | 12,86 | 10,38 | 13,57 | 0,02 |
| CTGF | 2,88 | 4,07 | 2,91 | 2,33 | -0,01 | 4,27 | 7,09 | 2,41 | 1,57 | 0,77 | 2,18 | 0,75 | 3,02 | 4,05 | -0,39 | 3,78 | 5,40 | 2,21 | 1,39 | 0,82 |
| CTSD | 0,72 | 1,00 | 0,59 | 0,34 | 0,23 | 1,19 | 1,36 | 0,52 | 0,63 | 1,31 | 1,70 | 1,70 | 0,51 | 0,53 | 2,35 | 0,96 | 1,05 | 0,53 | 0,88 | 0,84 |
| CXCR4 | 2,91 | 1,76 | 2,62 | 1,13 | 0,11 | 3,63 | 2,72 | 2,63 | 1,03 | 0,38 | 2,90 | 1,00 | 2,84 | 1,74 | 0,02 | 3,20 | 2,16 | 2,69 | 1,24 | 0,15 |
| EDN1 | 7,39 | 8,56 | 7,21 | 9,82 | 0,02 | 9,32 | 11,97 | 6,56 | 7,37 | 0,42 | 7,87 | 2,90 | 7,26 | 9,41 | 0,08 | 9,67 | 10,65 | 5,57 | 6,56 | 0,86 |
| EGLN1 | 3,17 | 4,41 | 3,22 | 3,75 | -0,01 | 4,89 | 7,07 | 2,60 | 2,66 | 0,88 | 4,74 | 3,57 | 2,89 | 4,32 | 0,64 | 4,53 | 5,71 | 2,21 | 2,50 | 1,17 |
| ENG | 1,27 | 1,20 | 1,18 | 1,08 | 0,08 | 1,74 | 1,92 | 1,08 | 0,74 | 0,62 | 1,51 | 1,00 | 1,20 | 1,19 | 0,26 | 1,68 | 1,58 | 0,94 | 0,61 | 0,87 |
| ENO1 | 1,50 | 1,60 | 1,95 | 1,94 | -0,31 | 2,17 | 2,77 | 1,38 | 1,08 | 0,57 | 1,39 | 0,41 | 1,63 | 1,80 | -0,18 | 2,23 | 2,35 | 1,10 | 0,44 | 1,20 |
| EPO | 23,83 | 34,89 | 41,84 | 65,41 | -0,76 | 41,99 | 50,93 | 22,75 | 40,77 | 0,85 | 42,13 | 60,60 | 26,03 | 41,60 | 0,62 | 42,45 | 56,70 | 12,74 | 18,51 | 2,72 |
| ERBB2 | 6,17 | 12,24 | 1,42 | 1,68 | 3,34 | 10,58 | 14,63 | 3,30 | 8,94 | 2,21 | 23,35 | 20,39 | 1,75 | 2,00 | 12,31 | 7,00 | 11,63 | 3,48 | 9,74 | 1,26 |
| ETS1 | 1,86 | 1,96 | 2,69 | 2,92 | -0,44 | 2,89 | 3,24 | 1,74 | 1,66 | 0,66 | 1,58 | 0,80 | 2,13 | 2,35 | -0,35 | 3,02 | 3,03 | 1,35 | 0,61 | 1,36 |
| FOXO3 | 1,14 | 1,36 | 1,25 | 1,07 | -0,09 | 1,71 | 2,13 | 0,98 | 0,82 | 0,73 | 1,50 | 1,08 | 1,10 | 1,33 | 0,36 | 1,62 | 1,71 | 0,88 | 0,97 | 0,86 |
| GLUT1 | 1,89 | 1,21 | 2,05 | 1,71 | -0,08 | 2,00 | 1,23 | 1,87 | 1,33 | 0,07 | 2,37 | 1,71 | 1,85 | 1,24 | 0,28 | 2,16 | 1,47 | 1,81 | 1,38 | 0,17 |
| GPI | 1,32 | 1,40 | 1,49 | 0,68 | -0,13 | 1,99 | 2,36 | 1,14 | 0,51 | 0,75 | 1,36 | 0,48 | 1,35 | 1,37 | 0,01 | 1,80 | 1,81 | 1,04 | 0,44 | 0,81 |
| IGF2 | 2,28 | 1,99 | 4,48 | 4,87 | -0,97 | 3,47 | 2,85 | 2,57 | 2,91 | 0,35 | 2,43 | 2,37 | 2,82 | 3,04 | -0,16 | 3,73 | 3,98 | 1,92 | 1,54 | 1,18 |
| KRT19 | 2,74 | 1,65 | 2,73 | 2,17 | 0,00 | 2,60 | 2,09 | 2,73 | 1,64 | -0,05 | 3,47 | 2,00 | 2,60 | 1,69 | 0,33 | 2,73 | 1,79 | 2,63 | 1,65 | 0,11 |
| LDHA | 1,24 | 0,76 | 1,20 | 0,37 | 0,03 | 1,36 | 1,02 | 1,16 | 0,56 | 0,17 | 1,52 | 0,57 | 1,18 | 0,70 | 0,29 | 1,56 | 0,84 | 0,96 | 0,37 | 0,76 |
| LEP | 1,61 | 2,10 | 2,28 | 4,51 | -0,41 | 1,47 | 1,64 | 1,82 | 2,91 | -0,24 | 2,39 | 3,50 | 1,62 | 2,53 | 0,48 | 1,10 | 1,39 | 1,98 | 2,92 | -0,53 |
| MET | 6,13 | 7,54 | 12,15 | 21,45 | -0,98 | 10,28 | 9,93 | 6,40 | 12,17 | 0,61 | 12,99 | 10,44 | 6,42 | 11,94 | 1,02 | 11,92 | 16,07 | 4,00 | 5,38 | 2,30 |
| MMP2 | 5,96 | 4,40 | 10,09 | 9,46 | -0,69 | 6,96 | 5,55 | 6,69 | 6,09 | 0,04 | 6,50 | 2,48 | 6,93 | 6,41 | -0,07 | 8,95 | 7,52 | 5,06 | 3,90 | 0,95 |
| NDRG1 | 3,88 | 3,76 | 4,15 | 3,08 | -0,07 | 4,89 | 4,55 | 3,57 | 3,19 | 0,37 | 5,16 | 5,72 | 3,72 | 3,15 | 0,39 | 4,20 | 3,46 | 3,59 | 3,30 | 0,26 |
| NHERF1 | 2,07 | 1,79 | 1,42 | 0,93 | 0,46 | 3,22 | 2,54 | 1,47 | 0,95 | 1,19 | 3,38 | 1,61 | 1,66 | 1,53 | 1,03 | 2,14 | 1,47 | 1,72 | 1,73 | 0,33 |
| NR4A1 | 5,19 | 5,84 | 3,85 | 3,04 | 0,35 | 4,27 | 3,92 | 5,03 | 5,69 | -0,18 | 4,16 | 2,75 | 5,03 | 5,72 | -0,21 | 4,81 | 4,78 | 4,83 | 5,84 | 0,04 |
| PGK1 | 2,41 | 2,78 | 2,09 | 1,65 | 0,15 | 3,86 | 4,58 | 1,82 | 1,13 | 1,11 | 3,21 | 1,57 | 2,18 | 2,69 | 0,47 | 3,19 | 3,58 | 1,70 | 1,09 | 1,00 |
| PLAUR | 4,01 | 6,39 | 5,18 | 5,28 | -0,29 | 6,64 | 11,50 | 3,48 | 3,18 | 0,91 | 3,60 | 1,20 | 4,42 | 6,66 | -0,23 | 6,85 | 8,97 | 2,68 | 2,03 | 1,63 |
| PTEN | 1,78 | 3,55 | 2,20 | 3,57 | -0,24 | 0,39 | 0,34 | 2,30 | 3,87 | -4,92 | 1,34 | 2,53 | 1,97 | 3,69 | -0,47 | 2,12 | 4,75 | 1,53 | 2,43 | 0,59 |
| SNAI1 | 2,82 | 4,12 | 3,16 | 3,30 | -0,12 | 4,45 | 7,06 | 2,35 | 2,03 | 0,90 | 2,94 | 1,63 | 2,89 | 4,22 | 0,02 | 4,23 | 5,57 | 1,88 | 1,28 | 1,45 |
| TGFB3 | 1,66 | 1,04 | 3,18 | 3,27 | -0,91 | 2,12 | 1,59 | 1,97 | 1,88 | 0,08 | 2,02 | 0,86 | 1,99 | 1,96 | 0,01 | 2,58 | 2,60 | 1,48 | 0,58 | 0,92 |
| TGM2 | 3,65 | 4,77 | 2,67 | 2,38 | 0,37 | 3,06 | 3,09 | 3,48 | 4,66 | -0,14 | 8,53 | 8,58 | 2,49 | 2,29 | 2,42 | 4,00 | 3,65 | 3,07 | 5,15 | 0,32 |
| TPI1 | 1,05 | 0,64 | 1,04 | 0,28 | 0,02 | 1,36 | 1,04 | 0,93 | 0,28 | 0,46 | 1,15 | 0,40 | 1,03 | 0,61 | 0,12 | 1,17 | 0,55 | 0,94 | 0,58 | 0,30 |
| TWIST1 | 7,78 | 9,52 | 6,56 | 5,56 | 0,19 | 9,64 | 11,25 | 6,69 | 7,75 | 0,44 | 4,69 | 1,08 | 8,04 | 9,44 | -0,71 | 7,10 | 8,04 | 7,26 | 9,93 | 0,09 |
| VEGFA | 0,66 | 0,55 | 1,00 | 0,91 | -0,51 | 0,72 | 0,81 | 0,72 | 0,59 | -0,01 | 0,67 | 0,51 | 0,74 | 0,67 | -0,11 | 0,97 | 0,83 | 0,56 | 0,43 | 0,80 |
| VHL | 1,09 | 0,98 | 1,44 | 1,34 | -0,32 | 1,48 | 1,62 | 1,06 | 0,79 | 0,40 | 1,07 | 0,49 | 1,18 | 1,13 | -0,10 | 1,59 | 1,47 | 0,86 | 0,42 | 0,93 |
| VIM | 2,13 | 2,71 | 3,05 | 3,32 | -0,43 | 3,31 | 4,69 | 2,00 | 1,86 | 0,65 | 1,88 | 0,40 | 2,41 | 3,07 | -0,28 | 3,28 | 4,08 | 1,56 | 0,68 | 1,31 |
